# Supplementary material for: The role of water in fault lubrication
Source: Nat Commun. 2018 Jun 13;9:2309. doi: 10.1038/s41467-018-04782-9 (PMC5998041; doi:10.1038/s41467-018-04782-9)
Supplement: Supplementary file 1 — Supplementary Information [file 41467_2018_4782_MOESM1_ESM.pdf]

Supplementary Information

The Role of Water in Fault Lubrication

Diao et al.

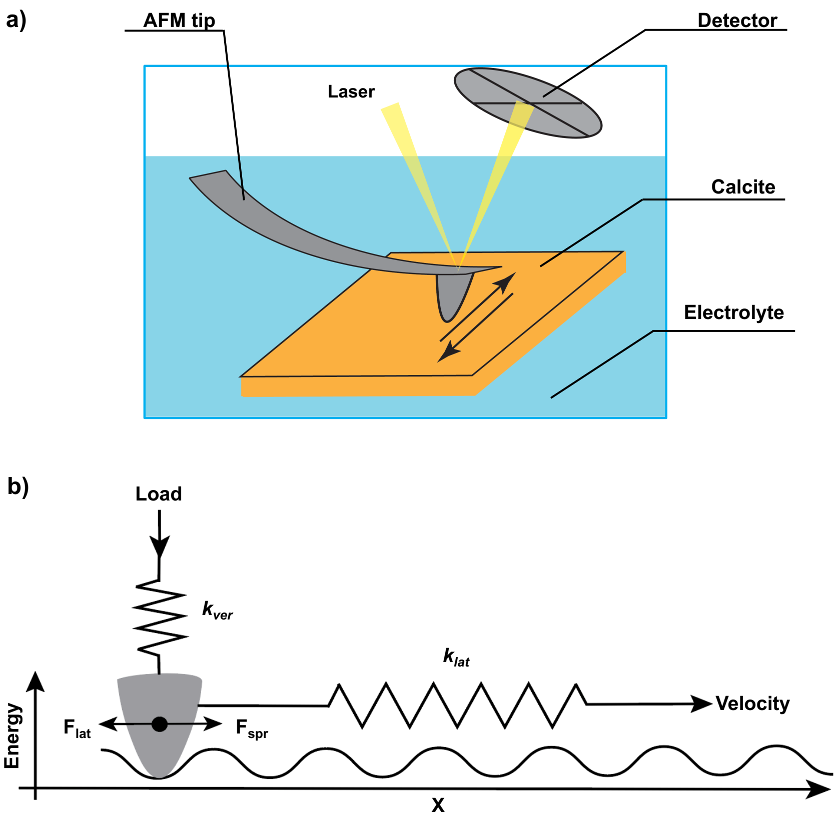

Supplementary Figure 1. Schematic description and mechanic configuration of the experimental set-up. a) Schematics of the calcite crystal immersed in the investigated electrolyte solution during the friction measurements. b) The AFM tip is driven by an internal piezo motor so that it slides 100 nm to the right and then to the left, thereby defining a friction loop, along the calcite surface at the selected constant velocity. The laser position reflected by the cantilever in a photodetector quantifies the vertical and the lateral deflection of the cantilever, and the force is determined with the corresponding normal and lateral spring constants (k_{vert} and k_{lat}). When the AFM tip slides along the calcite surface, it experiences a lateral force F_{lat}, which leads to a torsion of the cantilever. During friction-force measurements, the applied load in maintained constant by a feedback loop, while the lateral force is determined with the measured lateral deflection of the cantilever and the lateral spring constant.

Supplementary Table 1: Calculated normal stress, contact radius, and contact area for each applied load according to the Hertz model [1].

Stress regime	Tip radius [nm]	Load [nN]	Radius [nm]	Area [nm^2]	Max. normal stress [MPa]	Ave. normal stress [MPa]
Low	150	0.5	1.2	4.2	181	121
		1	1.4	6.6	230	153
		2	1.8	10.2	287	191
Intermediate	150	5	2.5	19.6	390	260
		10	3.1	30.2	492	328
		15	3.6	40.7	563	375
		20	3.9	47.8	620	413
High	100	10	2.7	22.9	644	429
		20	3.4	36.3	812	541
		30	3.9	47.8	930	620
		40	4.3	58.1	1023	682
		50	4.7	67.9	1100	733
Dry	100	10	2.7	22.9	644	429
		20	3.4	36.3	812	541
		30	3.9	47.8	930	620

Supplementary Table 2: Calculated ion concentrations, and calculated and measured pH values of the solutions.

$CaCl_2$	Ca^{2+} [mM]	Cl^- [mM]	CO_3^{2-}/HCO_3^- [mM]	Measured pH	Calculated pH
0 mM	0.51	0.00	1.02	8.30	8.23
1 mM	1.33	1.99	0.68	7.89	8.04
10 mM	9.90	19.74	0.33	7.58	7.69
100 mM	46.32	96.23	0.21	7.24	7.34
1 M	277.90	777.91	0.07	7.01	7.02

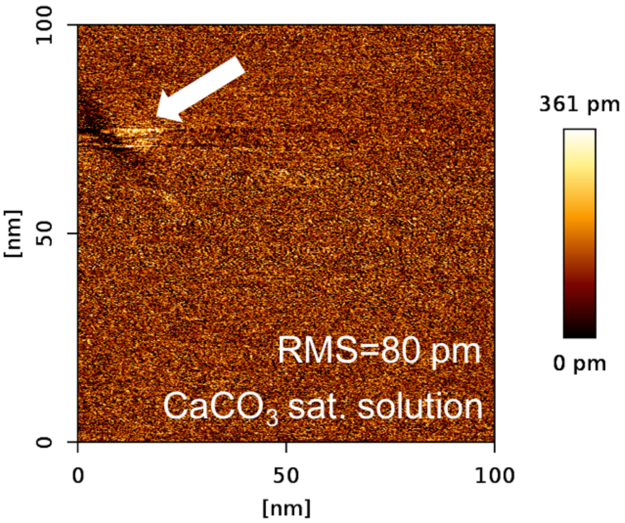

Supplementary Figure 2. AFM image of the (1014) calcite plane (area=100 nm*100 nm) equilibrated in saturated aqueous solution with calcite. The arrow points at the etching of the surface in the solution. Such regions were avoided in friction force measurements, which were only performed on atomically flat surfaces.

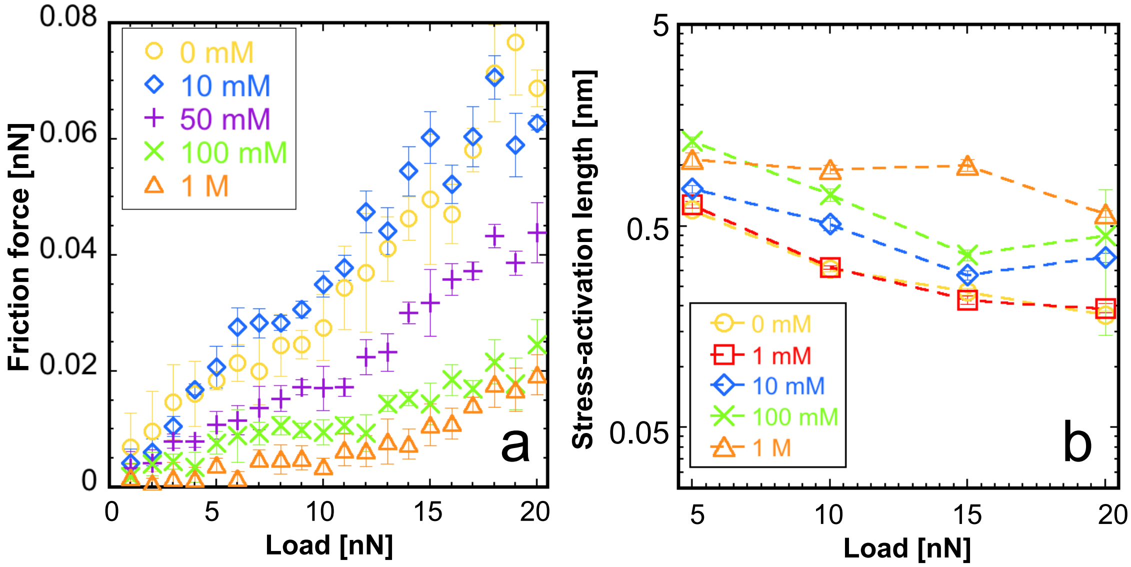

Supplementary Figure 3. Reference friction-force measurements conducted on mica in NaCl solutions with a silicon AFM tip. a) Friction force as a function of load at a sliding velocity of 0.2 μm s^{-1} at selected concentrations of NaCl (0 mM, 10 mM, 50 mM, 100 mM and 1 M). b) Shear activated length calculated from fits of Eq. 2 to the velocity-dependent friction force (not shown). The friction force as a function of the normal load decreases notably with increase in NaCl concentration, while the shear activated length increases with NaCl concentration as a result of the higher mobility of the confined hydrated ions, which facilitates sliding.

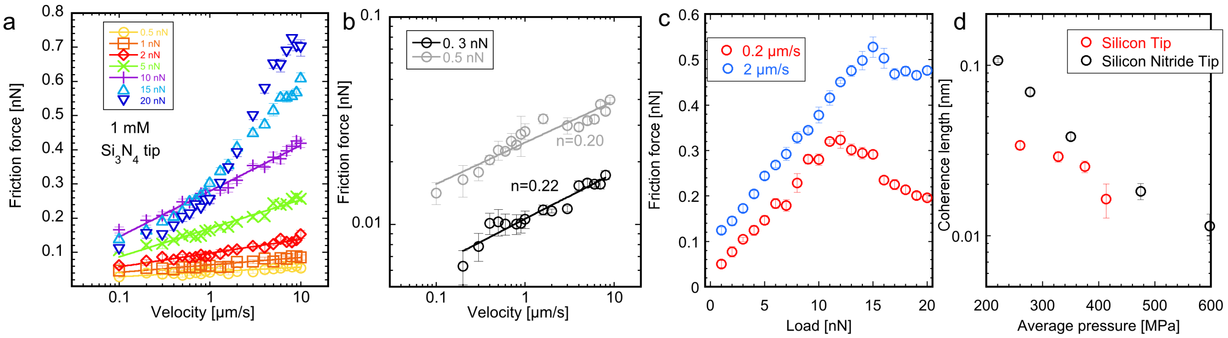

Supplementary Figure 4. a-b) Friction force between a silicon nitride tip and calcite in 1 mM $CaCl_2$ solution as a function of the sliding velocity. Although we chose silicon nitride tips with radii of 100 nm and 150 nm, the higher Young's modulus of silicon nitride compared to silicon leads to a higher average Hertzian stress compared to the results shown in the main text under the same applied normal loads. Therefore, lower loads were applied to evaluate the three regimes. c) Friction force as a function of load measured with the silicon nitride tip shows a transition corresponding to the onset of pressure solution of calcite. d) Comparison of the shear activated lengths obtained with silicon and silicon nitride tips in 1mM $CaCl_2$ solution.

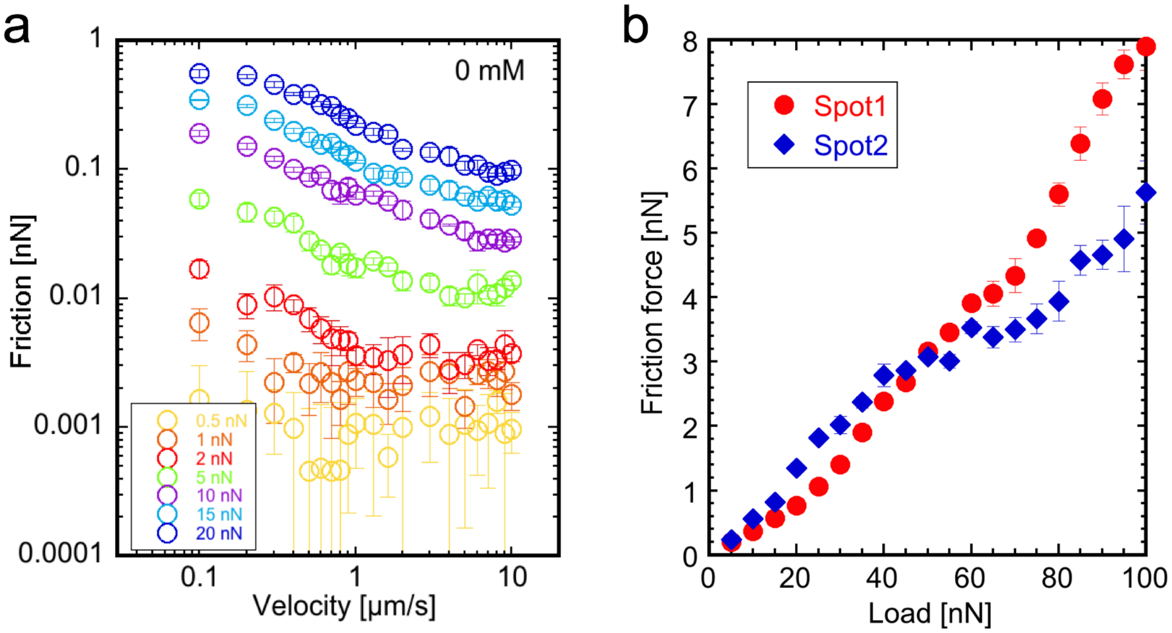

Supplementary Figure 5. Friction force between a (naturally oxidized) silicon wafer and a (naturally oxidized) silicon tip in water a) as a function of velocity and b) as a function of applied load at two different locations.

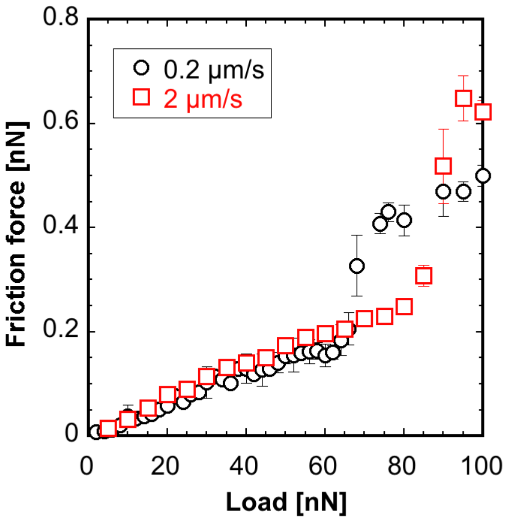

Supplementary Figure 6. Friction force as a function of load between the AFM tip and mica in water at two sliding speeds. No decrease of friction force is detected in the range of investigated loads. Instead, the friction force abruptly jumps up, likely caused by the squeeze-out of the water film. The hydration energy of mica is ~65±5 kJ mol^{-1} [2]. Given 0.27 nm as the size of a water molecule and the tip-substrate contact area (Table S2), the estimated work W applied by the tip (radius=30 nm) to push out a water monolayer adsorbed on mica is 71 kJ mol^{-1}, as shown in Table S3. This estimation supports that the abrupt increase in friction is caused by the removal of the monolayer of water adsorbed on mica at a load of ~60 nN. Mica is regarded as insoluble, i.e. the activation energy for mica dissolution is infinitely high, and indeed, no pressure solution of mica was ever observed in previous studies [3]. It should be noted that the hydration enthalpy of calcite is much higher than that of mica (94 kJ mol^{-1} [4]) and dehydration cannot happen under the pressures applied in this work (W =27 kJ mol^{-1} at the load of 50 nN with a tip radius of 100 nm).

Supplementary Table 3: Estimated work applied by the AFM tip assuming that it is used to remove a monolayer of water adsorbed to calcite and mica, respectively. The estimated work is calculated with $\mathrm{W\ (kJ/mol)} = \frac{F*\Delta*A_{mol}}{\pi a^2/4}$ according to ref. 5, where F is the applied load with the tip, a/2 is the Hertzian radius (Supplementary Table 2), $A_{mol} = N_A \pi \left(\frac{\Delta}{2}\right)^2$ is the area occupied by a layer of water molecules per mole, and Δ is the diameter of a water molecule (~0.27 nm).

Substrate	Load [nN]	kJ mol^{-1}
calcite	10	16.2
	20	20.5
	30	23.4
	40	25.6
	50	27.4
mica	60	71.1
	80	73.12

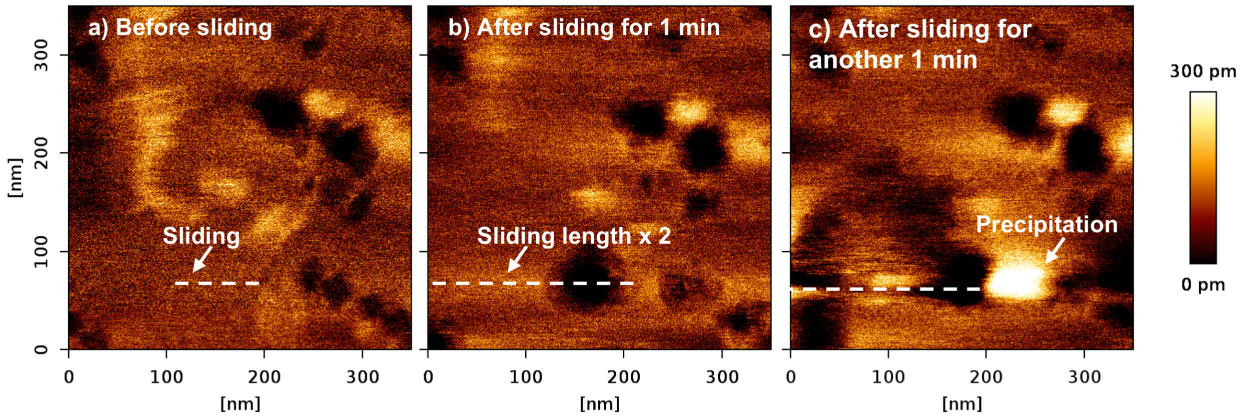

Supplementary Figure 7. AFM images of calcite surfaces equilibrated in 1 mM $CaCl_2$ before and after prolonged sliding at ~500 MPa showing the track of the AFM tip as a result of the pressure-induced dissolution of calcite. We note that after short sliding, like in the measurements shown in the main text, the track is not clearly visible at the selected scale (pressure applied for a time≤20 s), and therefore, we prolonged the sliding time to clearly demonstrate this phenomenon here. The dashed lines in Figures a-c) indicate where the sliding was conducted. After 1-min sliding, a track was detected. And a longer track was observed after sliding for another 1 min over a longer distance, while the topography changed nearby, which is attributed to the re-precipitation of dissolved calcite.

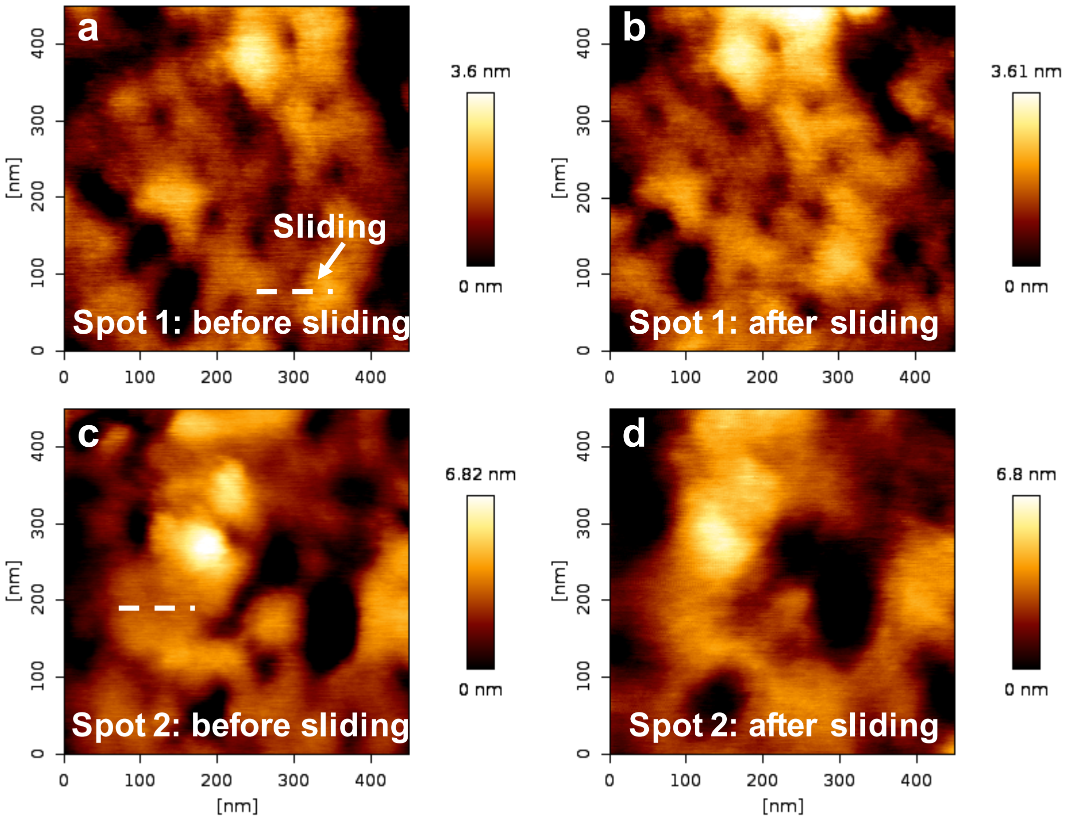

Supplementary Figure 8. AFM images of calcite surfaces equilibrated in ethanol before (a, c) and after (b, d) prolonged sliding at ~500 MPa (similar conditions as in Supplementary Figure 7). No visible track of the AFM tip is observed in either Spot 1 (a, b) or Spot 2 (b, d).

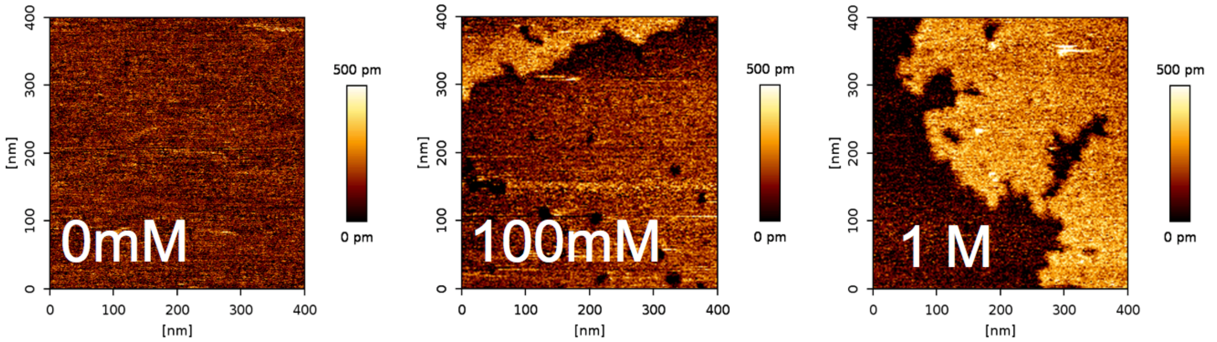

Supplementary Figure 9. AFM images of calcite surfaces equilibrated in $CaCl_2$ solutions (0mM, 100mM, 1M) showing an enhanced surface reconstruction with increase in concentration, which illustrates the higher reactivity of calcite.

Supplementary References

1 Johnson, K. L. in *Contact Mechanics* Ch. 4, (Cambridge University Press, 1985).
2 Leng, Y. & Cummings, P. T. Hydration structure of water confined between mica surfaces. *The Journal of Chemical Physics* **124**, 074711, doi:10.1063/1.2172589 (2006).
3 Alcantar, N., Israelachvili, J. & Boles, J. Forces and ionic transport between mica surfaces: implications for pressure solution. *Geochimica et Cosmochimica Acta* **67**, 1289-1304, doi:10.1016/s0016-7037(02)01270-x (2003).
4 de Leeuw, N. H. & Parker, S. C. Surface Structure and Morphology of Calcium Carbonate Polymorphs Calcite, Aragonite, and Vaterite: An Atomistic Approach. *The Journal of Physical Chemistry B* **102**, 2914-2922 (1998).
5 Diao, Y. & Espinosa-Marzal, R. M. Molecular insight into the nanoconfined calcite–solution interface. *Proceedings of the National Academy of Sciences* **113**, 12047-12052, doi:10.1073/pnas.1605920113 (2016).
